# Flexion contracture can cause component mismatch in the Prophecy^®^ preoperative patient-specific instrumentation for Evolution^®^ medial-pivot knee system

**DOI:** 10.1051/sicotj/2024009

**Published:** 2024-04-09

**Authors:** Shuhei Hiyama, Tsuneari Takahashi, Mikiko Handa, Katsushi Takeshita

**Affiliations:** 1 Department of Orthopedics, Jichi Medical University 3311-1 Yakushiji Shimotsuke Tochigi 329-0498 Japan; 2 Department of Orthopedics, Ishibashi General Hospital 1-15-4 Shimokoyama Shimotsuke 329-0502 Japan

**Keywords:** Prophecy Evolution medial-pivot patient-specific instrumentation (PSI), Total knee arthroplasty (TKA), Accuracy, Component match ratio, Medial pivot knee system

## Abstract

*Introduction*: Patient-specific instrumentation (PSI) systems are used to conduct total knee arthroplasty. PSI reduces operative time, is less invasive and easier to use, and minimizes the risk of errors by providing precise measurements and reducing operating room turnover time. However, a study on the accuracy of Prophecy Evolution PSI (Microport Inc., Arlington, TN, USA) reported that 94% were below the error margin of 1.5 mm and 90% had error margins of 1 mm. This study aimed to evaluate the accuracy of the Prophecy Evolution PSI system in terms of the thickness of “total” bony resection required to achieve adequate extension/flexion gaps and the component match ratio between preoperative planning and actual component size inserted. *Methods*: Comparisons were made between the sizes of femoral and tibial components planned with PSI and those inserted. The primary outcome was the average preoperative range of motion with and without matched femoral/tibial components. The study further analyzed the proportions of cases in which both the femoral and tibial components matched, neither matched, and only one of the femoral or tibial components matched. *Results*: The ratio of the same sizes between the PSI planning and those inserted was 50.8% (33 patients) for both the femoral and tibial components. For the femoral component alone, the ratio was 84.6% (55 patients), and for the tibial component, it was 58.4% (38 patients). A receiver-operating characteristic curve analysis indicated that flexion contracture greater than 20° was a significant prognostic factor for the PSI component match group versus the mismatch group. *Discussion*: Flexion contracture may cause PSI mismatch. Notably, flexion contracture greater than 20° was a significant risk factor for the PSI component match group versus the mismatch group. During preoperative planning for a patient with flexion contracture, surgeons should prepare for the possibility of inserting an undersized tibial component.

## Introduction

Patient-specific instrumentation (PSI) systems are used to conduct total knee arthroplasty (TKA) in patients lacking access to the intramedullary canal [1]. PSI reduces the duration of surgery and is less invasive. It is also easier to use and minimizes the risk of surgical errors by providing precise measurements, thereby reducing the turnover time in the operating room [2, 3]. Furthermore, PSI does not require the use of intramedullary rods to establish alignment. This avoids disturbing the intramedullary canal and potentially reduces the risk of intraoperative fat embolism, a complication reported to occur in 46%–65% of cases using traditional surgical methods [4–6]. The surgical plan, in combination with the cutting guides, determines the resection thickness, component size, femoral rotation, and alignment of the femoral and tibial components. Accurate preparation of the femoral and tibial surfaces is critical for component positioning and, consequently, alignment/rotation, which affects function and longevity [7]. Based on evidence from systematic reviews and a meta-analysis of randomized clinical trials, there were no clinically relevant differences in efficacy and accuracy between patients treated with PSI TKA and non-PSI TKA [8–10]. PSI technology for the performance of TKA has developed rapidly, showing promising results from some perspectives and inconsistent results from other perspectives. Care must be taken to draw appropriate conclusions from the results of previous studies because the issue is complex and has many facets that may be interpreted differently [11]. Although one study on the accuracy of Prophecy Evolution PSI (Microport Inc., Arlington, TN, USA) reported that 94% were below the error margin of 1.5 mm and 90% had error margins of 1 mm when compared with the original surgical plan [12], the utilization of PSI did not reduce the number of outliers in the sagittal and coronal alignment of the tibial component [13]. Furthermore, no studies have reported whether the amount of bone resection achieved adequate extension/flexion (Ext/Flex) gaps in TKA using PSI alone or compared preoperative planning with the actual component sizes inserted. Ligament balance cannot be predicted preoperatively on CT images, necessitating careful soft tissue release in some cases [12]. This study aimed to evaluate the accuracy of the Prophecy Evolution medial-pivot PSI knee replacement system in terms of the thickness of “total” bony resection required to achieve adequate Ext/Flex gaps and the component match ratio between preoperative planning and the actual component sizes inserted.

## Materials and methods

### Patient enrollment

This retrospective study was conducted in the Department of Orthopedic Surgery at a single institution. The institutional review board of the ethics committee at the institution approved the study and waived the requirement for formal written informed consent, given the retrospective nature of the study. The ethics number is 23-065.

The inclusion criteria for the study were consecutive patients with osteoarthritis of the knee who underwent TKA using the PSI system. Data were collected from January 2019 to August 2023 for retrospective analysis. During this period, all patients who underwent TKA using PSI were included, and there were no excluded cases. The data for the patients were collected from an electronic medical database.

Preoperative hip–knee angle (HKA) and range of motion (ROM) for both extension and flexion were evaluated. The HKA was defined as (+) for varus rotation and (−) for valgus alignment. Comparisons were made between the sizes of femoral and tibial components planned with PSI and those actually inserted. The primary outcome was the average preoperative ROM with and without matched femoral/tibial (*F*/*T*) components. This “match” refers to the congruence between the actual sizes of the femoral and tibial components and their respective sizes planned preoperatively with PSI. The study also analyzed the proportions of cases in which both femoral and tibial components matched, neither matched, and only one of the femoral or tibial components matched. The total thickness of the distal femoral, dorsal femoral, and proximal tibial osteotomies were compared between preoperative planning and actual surgery. In addition, the total thickness of the tibial re-cut was compared between cases with and without flexion contracture. The thickness of the bone cut was measured using calipers.

### Surgical procedure

All TKA procedures were performed using a Prophecy Evolution system (MicroPort Orthopedics Inc., Arlington, TN, USA) with PSI, comprising a cemented, fixed-bearing implant, following the medial parapatellar approach. In all cases, both the anterior cruciate ligament and posterior cruciate ligament were dissected. Patella resurfacing was not performed. Infrapatellar fat pad excision was only conducted when necessary for adequate surgical visualization and was minimized to prevent anterior knee pain [14].

The TKA with PSI technique employs a measured resection technique, initially creating an Ext gap [15]. Conversely, the bony cut uses a measured resection technique with PSI based on bony landmarks. The process began with a distal femoral cut using PSI guides, followed by a proximal tibial cut to achieve a suitable Ext gap, as per the preoperative plan. If the Ext gap was insufficient post-resection, the proximal tibial was re-cut by 2-mm increments without soft tissue release and an implant one size smaller was inserted. Once the Ext gap was suitable, the dorsal femoral cut was performed at a knee flexion of 90° using PSI guides. A similar approach was applied if the Flex gap was too narrow and included re-cutting the dorsal femur by 2 mm without soft tissue release. After the final bone resections, the Ext/Flex gaps were assessed. Throughout the procedure, the personalized cutting guides functioned either as slotted guides for primary femoral distal and proximal tibial cuts or as precise pin positioning aids. We avoided any gap-balancing techniques that involved releasing the collateral or retinacular ligaments to adjust the Ext/Flex gaps. The preoperative PSI plan included a surgical saw thickness allowance of up to 1.28 mm, which was taken into account in thickness comparisons. Careful incision and balancing of soft tissues were crucial, maintaining osteophytes as reference points for guide placement.

### Statistical analysis

Data are presented as mean and standard deviation (SD). An *a priori* power analysis was performed using G*Power 3.1 (Franz Paul, Kiel, Germany) [16]. To assess the normality of the data, we conducted a comprehensive evaluation using three methods: histograms, QQ plots, and tests for normality. Based on these assessments, we decided to use a paired *t*-test for our analysis. The sample size for the paired *t*-test, targeting the primary outcome, was assessed *a priori*, with the significance threshold set at *P* < 0.05. The minimum sample size required, based on an *α* error of 0.05, a *β* error of 0.20, and Cohen’s effect size of 0.8 with an allocation ratio of 1, was determined to be 24 patients. The component match group included 33 patients, and the component mismatch group included 32 patients. A post hoc analysis revealed a *β* error of 0.06 (i.e., power of 0.94) with an effect size of 0.8. An *α* error of 0.05 was considered statistically significant. All statistical analyses were performed using EZR software (http://www.jichi.ac.jp/saitama-sct/SaitamaHP.files/statmed.html) [17]. A receiver-operating characteristic (ROC) curve analysis was also performed.

## Results

**Figure 1:**
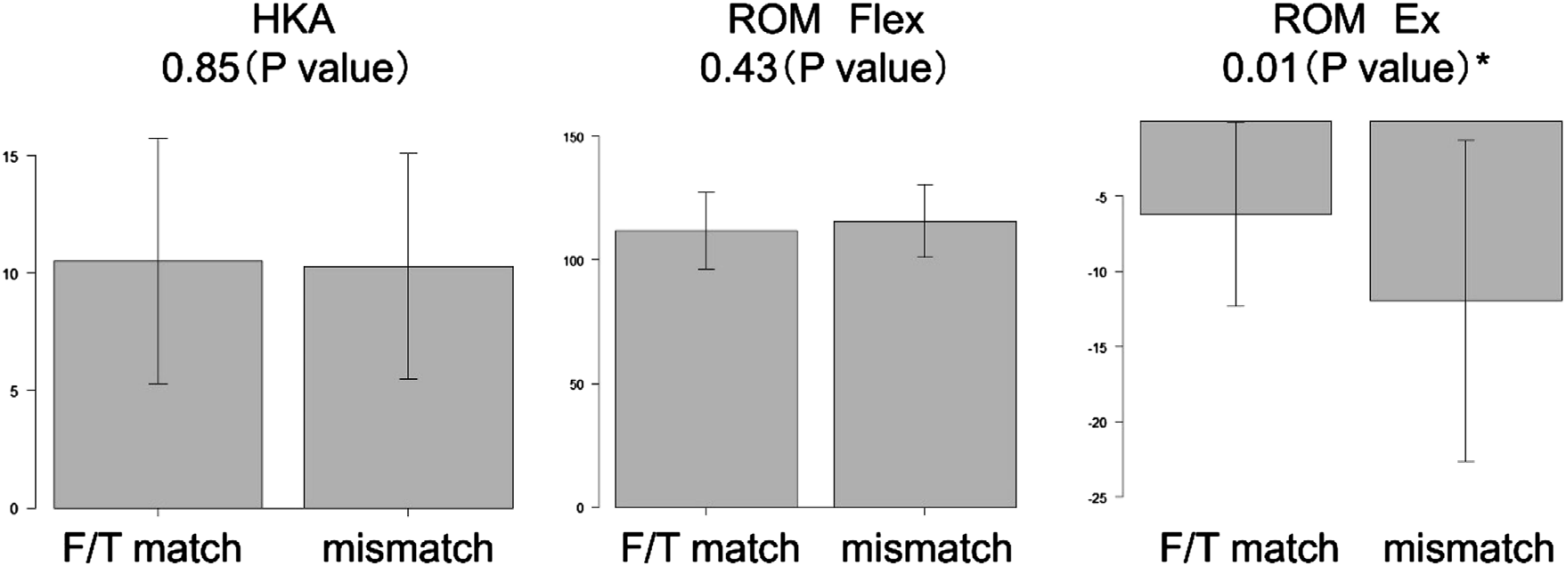
Comparisons of preoperative parameters between the femoral/tibial (*F*/*T*) match and mismatch groups. ^*^Student’s *t*-test.

The study included 65 patients with a mean age of 72.9 years (SD: 8.5; minimum-maximum [min-max]: 54–83) years who underwent PSI-assisted TKA. The preoperative mean HKAs and ROMs for extension and flexion were 10.4° (SD: 5.0; min-max: 1–22.3), −9.1° (SD: 9.0; min-max: −45–0), and 114.6° (SD: 15.0; min-max: 50–135), respectively. The ratio of the same sizes between the PSI planning and those inserted was 50.8% (33 patients) for both the femoral and tibial components. For the femoral component alone, the ratio was 84.6% (55 patients), and for the tibial component, it was 58.4% (38 patients). Regarding the femoral component, 3.1% (2 patients) received an insert one size over, 10.8% (7 patients) one size under, and 1.5% (1 patient) a half size under. For the tibial component, 36.9% (24 patients) received an insert one size under, and 4.6% (3 patients) a half size under. The average HKAs for the *F*/*T* match and no-match were 10.5° and 10.3°, respectively, with no significant difference. The average flexion was 113.2° in the *F*/*T* match group and 116.1° in the no-match group. No significant difference was noted. Meanwhile, the average extension was −6.1° in the *F*/*T* match group and −12.2° in the no match group, and the difference was significant (*P* < 0.05) (Figure 1).

**Figure 2:**
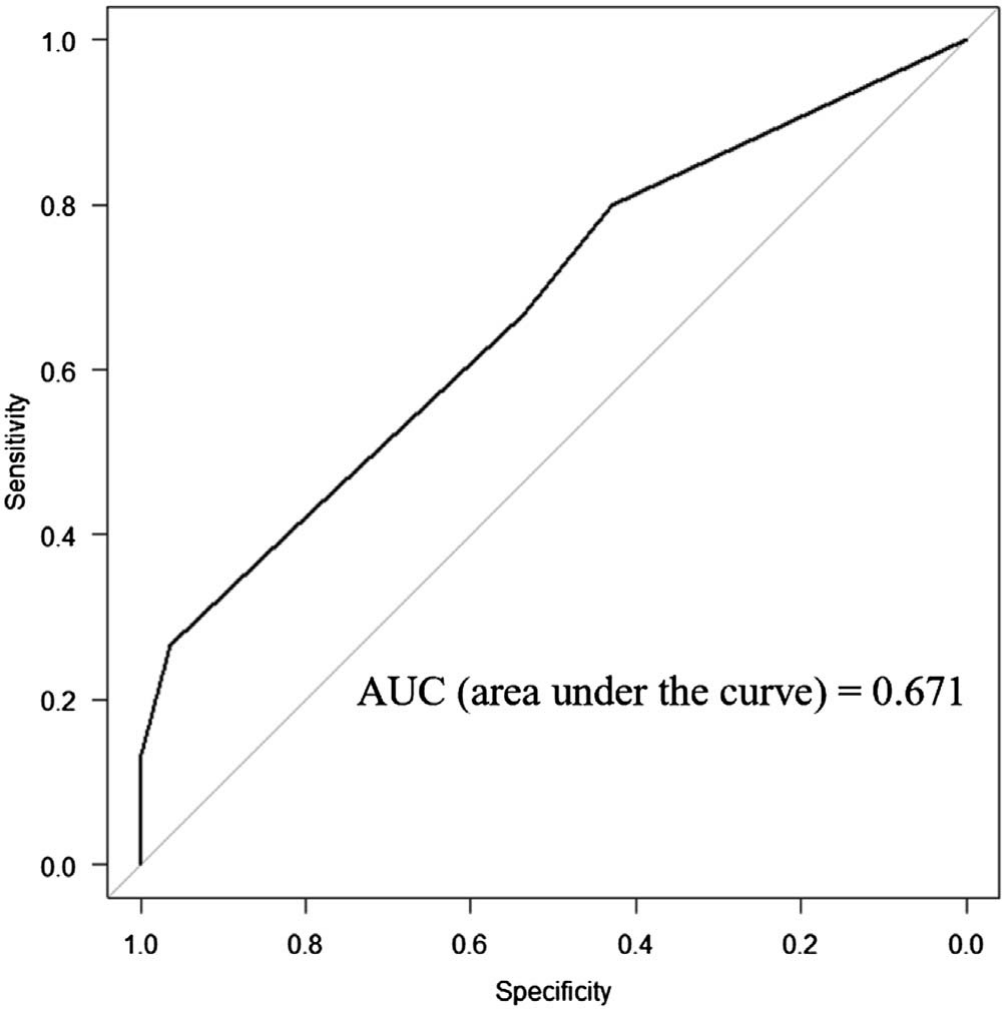
Receiver-operating characteristic curve of flexion contracture for patient-specific instrumentation mismatch cases.

The ROC curve analysis indicated that flexion contracture greater than 20° was a significant prognostic factor for the PSI component match group versus the mismatch group (odds ratio: 9.82; 95% confidence interval: 1.14–84.6; *P* < 0.05) (Figure 2).

When the flexion contracture cutoff value of 20° was applied to our patient cohort, the sensitivity, specificity, positive predictive value (PPV), and negative predictive value (NPV) of the PSI match ratio was estimated to be 28.1%, 97.0%, 90.0%, and 49.2%, respectively (Table 1).

**Table 1:** Numbers of patient-specific instrumentation match and mismatch cases with and without flexion contracture.

	PSI match	PSI non-match	*P* value*
Flex cont. (+)	1	9	<0.05
Flex cont. (−)	32	23	<0.05

* Fisher’s exact test.

Flex cont.: flexion contracture.

Average differences of 1.0 mm and 0.1 mm were observed in the thickness of the distal femur bone resection for the medial and lateral sides, respectively. A significant difference (*P* < 0.01) was observed on the medial side. On average, discrepancies of 1.8 mm and 1.7 mm were observed in the thickness of bony resection of the dorsal femur for the medial and lateral sides, respectively. Significant differences (*P* < 0.01) were noted on both sides. In the femur, bony resection tended to be less than the PSI prediction. Average discrepancies of 2.4 mm and 3.2 mm were observed in the thickness of bone resection of the proximal tibial for the medial and lateral sides, respectively. Significant differences (*P* < 0.01) were observed for both sides. In the tibial, bone resection tended to be greater than the PSI prediction (Table 2).

**Table 2:** Comparisons of the thicknesses of bone resection between PSI planning and actual measurements.

	PSI (mm)	Actual measurement (mm)	*P* value*
Distal femur			
Medial	9.6 (1.3)	9.9 (1.7)	<0.001
Lateral	7.7 (1.7)	8.8 (2.1)	0.4
Dorsal femur			
Medial	12.5 (1.4)	11.9 (2.2)	<0.001
Lateral	10.1 (1.5)	9.7 (1.9)	<0.001
Proximal tibial			
Medial	2.0 (1.4)	5.7 (2.4)	<0.001
Lateral	6.8 (1.7)	11.5 (2.9)	<0.001

* Comparisons between groups were made using a paired *t*-test.

Data are expressed as the average (standard deviation). The actual measurements included a surgical saw thickness of up to 1.28 mm.

The amounts of tibial re-cut with and without flexion contracture were 0.64 (1.46) mm and 1.80 (1.75) mm, respectively, for the group with flexion contracture. The differences in the amount of tibial re-cut between the groups with and without flexion contracture were significant.

When the flexion contracture cutoff value of 20° was applied to our patient cohort, the sensitivity, specificity, PPV, and NPV of the tibial re-cut ratio were estimated to be 35.2%, 91.7%, 60.0%, and 80.0%, respectively, for the number of tibial re-cut cases (Table 3).

**Table 3:** Numbers of tibial re-cut cases with and without flexion contracture.

	Tibial re-cut (+)	Tibial re-cut (−)	*P* value*
Flex cont. (+)	6	4	<0.05
Flex cont. (−)	11	44	<0.05

* Fisher’s exact test.

Flex cont.: flexion contracture.

## Discussion

In our study, the ratio of the same size between the PSI planning and the inserted components was 84.6% for the femoral component and 58.4% for the tibial component. For the tibial component, 36.9% of patients received a component one size under, while the remaining 4.6% received a component half a size under. Marchand et al. [18] reported that the accuracy of preoperative CT scan 3D templating was 81% for the femoral component and 80% for the tibial component, with all cases being within one size of the planned components (100%). The largest study to date in the literature, which evaluated the accuracy of 3D cutting blocks, aimed to determine the accuracy of actual intraoperative resections versus proposed resections [12]. However, there are no reports on the ratio of component match and accuracy with CT-based PSI in TKA. This report is the first to detail the accuracy of the component match ratio using the Prophecy Evolution medial-pivot PSI knee replacement system. Our study found that the component match ratio of the same size between PSI planning and the inserted components was 84.6% for the femoral component and 58.4% for the tibial component. The match ratio for the femoral component was similar to that of 3D templating, whereas the match ratio for the tibial component was lower. One plausible reason for the lower match ratio in the tibial component was the need to add tibial bony resection to obtain an adequate Ext gap. We employed an extension-first measured resection technique; after creating an adequate Ext gap, the dorsal femoral cut was performed with the knee in 90° of flexion using PSI guides. If the Flex gap was too small after resection, the dorsal femoral cut was re-performed with an adjustment of 2 mm, without soft tissue release. We observed average discrepancies of 4.4 mm and 10.2 mm in the thickness of bone resection of the proximal tibial on the medial and lateral sides, respectively, with significant differences (*P* < 0.01) on both sides. Flexion contracture may contribute to PSI mismatch, particularly when the flexion contracture exceeds 20°, a significant prognostic factor for the PSI component match group versus the mismatch group. When applying a flexion contracture cutoff value of 20° to our patient cohort, the number of tibial re-cut cases and the amount of re-cut with flexion contracture were more than those without flexion contracture.

Tibial components that were one size or a half size smaller than planned in PSI were inserted in 41.5% of the patients. During preoperative planning for a patient with extension contracture, surgeons should anticipate the need to insert an undersized tibial component.

Previous studies on the accuracy of osteotomy using patient-specific cutting guides in TKA have indicated that an acceptable difference between planned and actual bone cuts is 1.5 mm or less [19]. The difference between planned and actual resected bone thicknesses for osteotomy has ranged from 0.5 to 2 mm with the use of patient-specific 3D cutting guides [20, 21]. In a series of 81 knees, Levy et al. [19] demonstrated that the difference was within acceptable limits for approximately 80% of femur cuts and 70% of tibial cuts, suggesting that patient-specific cutting guides are moderately accurate. Our findings also indicated that the osteotomy error was within acceptable limits for all planes, highlighting the high accuracy of 3D patient-specific cutting guides. Additionally, the surgeon’s CT-based implant plan aligned within one size of the utilized implant 100% of the time for both tibial and femoral components. On a per-case basis, PSI systems may offer cost savings related to implants compared with the overall institutional cost for standard TKA, owing to reduced instrument preparation for only the same size and one size smaller components [22]. The PSI approach also saves surgical time. This time efficiency can result in significant financial benefits for high-volume tertiary centers and may have a broader impact on the healthcare system.

While PSI is garnering increasing scientific and practical interest, numerous authors have reported improved alignment and component positioning [23, 24]. According to Dorling et al. [22], when considering the total cost per patient case, PSI TKA is more expensive than conventional instrumentation TKA. This higher cost is attributed to the imaging and production expenses associated with PSI TKA, despite it being less expensive than conventional instrumentation TKA in terms of mean operating room time, associated costs, and tray sterilization per patient case. However, PSI systems can be cost-effective if their imaging and production costs can be decreased. PSI may be more attractive than computer-navigated or robotic surgeries because the latter require additional and costly time for registration [2, 3, 25]. Conversely, there are also reports showing no benefit or even unsatisfactory results for PSI [26].

This study is the first to report the precision of the component match ratio in the Prophecy Evolution medial-pivot PSI knee replacement system. However, our study has several limitations. The primary limitations of the study are its small sample size and noncomparative design, which prevent us from drawing more decisive conclusions regarding the advantages and disadvantages of using patient-specific cutting guides during TKA. In addition, we did not evaluate the clinical outcomes of TKA. Based on our findings, further large-scale studies with longer follow-ups comparing patient-specific cutting guides with conventional methods are needed to reach a more definitive conclusion about the efficiency of 3D patient-specific cutting guides for TKA.

In conclusion, flexion contracture may cause PSI mismatch. Notably, flexion contracture greater than 20° was a significant risk factor for the PSI component match group versus the mismatch group. During preoperative planning for a patient with flexion contracture, surgeons should prepare for the possibility of inserting an undersized tibial component.

## Data Availability

Data are available on request from the authors.
